# Exploring Unconventional Risk-Factors for Cardiovascular Diseases: Has Opioid Therapy Been Overlooked?

**DOI:** 10.3390/ijerph16142564

**Published:** 2019-07-18

**Authors:** Oluwabunmi Ogungbe, Luma Akil, Hafiz A. Ahmad

**Affiliations:** 1 Department of Epidemiology and Biostatistics, School of Public Health, Jackson State University, Jackson, MS 39213, USA; 2 Department of Behavioral and Environmental Health, School of Public Health, Jackson State University, Jackson, MS 39213, USA; 3 Department of Biology, Jackson state University, Jackson, MS 39217, USA

**Keywords:** cardiovascular diseases, opioids medication, hypertension, myocardial infarction

## Abstract

Approximately 2150 adults die every day in the U.S. from Cardiovascular Diseases (CVD) and another 115 deaths are attributed to opioid-related causes. Studies have found conflicting results on the relationship between opioid therapy and the development of cardiovascular diseases. This study examined whether an association exists between the use of prescription opioid medicines and cardiovascular diseases, using secondary data from the National Hospital Ambulatory Medical Care Survey (NAMCS) 2015 survey. Of the 1829 patients, 1147 (63%) were male, 1762 (98%) above 45 years of age, and 54% were overweight. The rate of cardiovascular diseases was higher among women [(*p* < 0.001), 95% CI: 0.40–0.51]. The covariates were age, race/ethnicity, sex, diabetes mellitus, hyperlipidemia, and hypertension; and were adjusted. Diabetes mellitus, hyperlipidemia, and hypertension were significant predictors of CVD [(*p* < 0.001, 95% CI: 0.57–0.78); (*p* < 0.001, 95% CI: 0.34–0.44); (*p* < 0.001, 95% CI: 0.49–0.59)]. There was no significant association between prescription opioid medication use and coronary artery disease [first opioid group *p* = 0.34, Prevalence Odds Ratio (POR): 1.39, 95% CI: 0.71–2.75; second opioid group: *p* = 0.59, POR: 1.20, 95% CI: 0.61–2.37, and third opioid group: *p* = 0.62, POR: 0.85, 95% CI: 0.45–1.6]. The results of this study further accentuate the conflicting results in literature. Further research is recommended, with a focus on those geographical areas where high prevalence of cardiovascular diseases exists.

## 1. Introduction

Cardiovascular diseases are the leading causes of death in the United States with 166 deaths per 100,000 in 2016. Thus, heart disease accounts for every one in four deaths in the U.S. [1]. There are several known risk factors for heart diseases, these are: hypertension, obesity, and diabetes, which are the classic triads that substantially increases the risks of cardiovascular diseases. Other risk factors include family history, behavioral factors such as, smoking, alcohol consumption, physical inactivity, and unhealthy diet. Some biological markers have also been indicated as risk factors, these are: elevated levels of C-Reactive Protein (CRP), low-density lipoproteins, high-density lipoproteins, triglycerides, homocysteine, etc. [2].

There are several recent studies on non-traditional risk factors of heart disease, one of such is long-term prescription opioid use due to substantial increase in the opioid use in the United States. In 2015, healthcare providers wrote opioid prescriptions at an average rate of 70 per 100 persons. All opioids have the potential for psychological dependence and about one in four adults who take opioid prescriptions for non-cancer pain struggle with lifelong addiction [3].

Although, long-term opioid therapy is commonly associated with adverse health outcomes, such as addiction and dependence, accidental overdoses, and sexual dysfunction, some recent studies have found links between prescription opioid drug use and cardiovascular diseases, especially myocardial infarction [4,5,6,7,8]. Other studies, however, suggest that opioids may have protective effects, and advocates for use of certain opioid drugs as cardioprotective agents [9,10].

Aghadavoudi, Eizadi-Mod, and Najarzadegan (2015), compared the levels of certain biochemical markers important in cardiovascular health such as low-density lipoproteins and free triglycerides in Coronary Artery Bypass Graft (CABG) candidates who are long-term opium users and those who are not. They found that the prevalence of coronary heart disease, hypercholesterolemia, and diabetes was higher amongst long-term opium users compared to non-users, after adjusting for intervening variables. The biochemical markers mentioned above were also higher among the opium user group [11]. A study in the Middle East region where long-term opioid use is prevalent, also found opioid drugs to be independent risk factors for coronary heart disease [12].

This issue remains a controversial one, and with the high prevalence of cardiovascular disease, and deaths from heart disease, it is important to explore both unknown and unconventional risk factors for heart disease. This is especially necessary considering the upsurge trend in the rate of prescription opioid use in many states. Opioid medications have always been used in medicine for the acute treatment of cardiovascular event such as Myocardial Infarction (MI), however, the contributions of use of these medications to cardiovascular morbidity have not been adequately explored. Thus, the objective of this study is to examine the association between use of prescription opioid medicines and cardiovascular diseases.

## 2. Materials and Methods

### 2.1. Study Design

This study is based upon analysis of secondary data obtained from the 2015 National Ambulatory Medical Care Survey (NAMCS). National Ambulatory Medical Care Survey and the National Hospital Ambulatory Medical Care Survey (NHAMCS) are annual national probability samples of ambulatory visits to emergency rooms, outpatient departments and short-stay hospitals in the United States, to non-federal, general and short-stay hospitals. The survey is conducted by the Centers for Disease Control and Prevention (CDC). A complete description of the data and methodology can be obtained elsewhere [13].

### 2.2. Participants

All respondents who reported to have Coronary Artery Disease (CAD) or MI are considered to have cardiovascular disease. The International Classification of Disease ICD 10 was used to encode for the diagnosis in the survey. ICD-10 code 125.1 encoded for atherosclerotic heart disease of the native coronary artery, this applies to coronary artery disease. ICD-10 code 120-125 codes for ischemic heart diseases, this includes acute Myocardial Infarction, including ST elevation MI (STEMI), and Non-ST elevation MI (NSTEMI) [14].

Definitions: coronary artery disease is the narrowing or blockage in the coronary arteries (arteries that supply the heart), due to atherosclerosis of these arteries. Myocardial infarction is the acute ischemic change that results from the sudden cut-off of blood supply form certain parts of the heart.

### 2.3. Variable Definitions

Coronary Artery Disease was defined as a self-reported history of CAD documented by a doctor or other healthcare professional in the past 12 months. Myocardial Infarction was defined as a self-reported history of MI documented by a doctor or other healthcare professional in the past 12 months. These were coded into variable CAD. Controlled substance code was used to record narcotics and opioid-containing medications. A 30-count survey medication list was used to encode and record the medications used.

Covariates included were patients’ age (recode), patient’s sex, race/ethnicity (imputed), smoking status (tobacco use, prior tobacco use), dyslipidemia, sedentary lifestyle, overweight/obesity, metabolic syndrome, diabetes mellitus (type 2), alcohol consumption and history of hypertension. Substance abuse or dependence was also included. Smoking status was classified as not-current; never, former or current smoker.

Body mass index (BMI) was calculated from actual measurements recorded. This was recoded into six groups: 1 = underweight (<18.5 kg/m^2^), 2 = normal weight (18.5–24.9 kg/m^2^), 3 = overweight (25.0–29.9 kg/m^2^), 4 = class 1 obesity (30.0–34.9 kg/m^2^), 5 = class II obesity (35.0–39.9 kg/m^2^), 6= class III obesity (≥40.0 kg/m^2^). History of chronic conditions was reported by asking “Does the patient now have…?” regardless of the diagnosis recorded for that visit. For Diabetes; “does the patient now have diabetes?” For hypercholesterolemia: “does the patient now have hypercholesterolemia?” for hyperlipidemia: does the patient now have hyperlipidemia?” For hypertension: “does the patient now have hypertension?”, and for obesity: “does the patient now have obesity?”

### 2.4. Medication Use

Prescribed use of controlled substances was coded in the NAMCS data according to the Controlled Substance Act (CSA) classification. The variable ‘CONTSUB’ 1–30 was derived from the medication list MED 1 to 30, which contained medicines that patients currently used. The CSA schedule which classifies, drugs and other substances which are considered controlled and of high abuse potential into five schedules in order of their potential for abuse and dependence. Schedule I substances are not currently used in medical practice, examples are heroin, methaqualone etc.

The data for schedule I was classified for research only and was not included in the public data used for this study. Schedule II substances have high abuse potential, with severe physical and psychological abuse. These drugs make up the majority of prescription opioid drugs used in medical practice, examples include hydromorphone (Dilaudid), oxycodone (Percocet) etc. Schedule III have a lesser abuse potential, e.g., Tylenol/codeine etc. Schedule IV substances also have a low potential for abuse, examples are alprazolam, clonazepam etc. Schedule V has a lower potential abuse, examples are Robitussin, codeine-containing cough preparations [15].

The NAMCS data coded these medications as ‘=‘ Blank’ ‘1’ =‘ Schedule I (Research only)’ ‘2’ = ‘Schedule II’ ‘3’ = ‘Schedule III’ ‘4’ = ‘Schedule IV’ ‘5’ = ‘Schedule V’ ‘6’ = ‘No control’ ‘7’ = ‘Undetermined’ ‘8’ = ‘Multiple schedules’. For the purpose of analyses, the groups were collapsed into two groups only: schedule II that is the reference group, and all other groups were combined into a second group.

### 2.5. Statistical Analyses

Statistical analyses were conducted using SPSS, version 25 (IBM, Armonk, NY, USA). The chi-square test for independence was used to determine whether difference exists between known risk factors of CVD (covariates) among patients who reported the disease (coronary artery diseases and myocardial infarction). Another set of chi-square test was performed to observe an initial association between the variables CONTSUB 1–30 and variable CAD.

Multivariable models for each outcome was fitted by adjusting for sociodemographic characteristics and the covariates. Age, sex, race, smoking status, overweight/obesity (BMI: ≥ 25 kg/m^2^), history of dyslipidemia, type 2 diabetes, hypertension was adjusted for. For each case, the dependent variable was CVD and the independent variables were CONTSUB 1, 2, and 3, which was found to be significant from the chi-square tests.

To determine whether the associations varied by opioid use, effect measure modification of covariates was tested by creating an interaction term of the categories of the variables. The interactions terms were interpreted at a significance level of *p* < 0.05.

## 3. Results

### 3.1. Sample Characteristics

A total of 1829 patients who were reported to have coronary artery disease and myocardial infarction were included in the study. This represented 6.5% of the total patients in the 2015 NAMCS survey. Sociodemographic characteristics of the study population are reported in Table 1 below. Of the 1829 patients, 1147 (63%) were male, and 1762 (97.9%) are above 45 years of age. Among the patients, 189 (10.3%) are current smokers, and 645 (35.3%) are former smokers. More than half of the sample—53.8% were either overweight or obese. Socio-economic data on income status, and education level could not be obtained from the data.

### 3.2. Cardiovascular Risks

**Figure 1:**
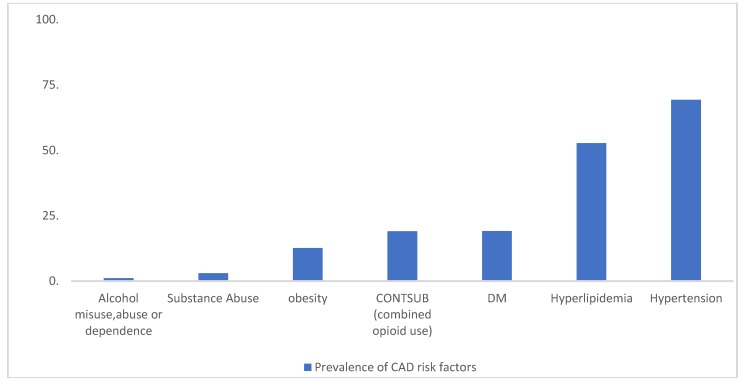
Prevalence of selected risk factors of CAD from the 2015 NAMCS data. Date source: Centers for Disease Control and Prevention, Center for Diseases Control and Prevention. National Ambulatory Health Care Data: Data Sets and Documentation. (November 30, 2017). Accessed September 27, 2018. https://www.cdc.gov/nchs/ahcd/datasets_documentation_related.htm [13].

Risk factors that were significantly associated with coronary artery diseases, from the chi-square test of independence are presented in Table 2 and illustrated in Figure 1.

### 3.3. Use of Prescription Opioid Medications

For the medication lists of 1 to 30 contained in the surveys, prescription opioid medications were combined into one group, and 19.1% of patients who had CAD routinely used these medications. The opioid medications mostly used by participants were acetaminophen-oxycodone, oxycodone, morphine, amphetamine-dextroamphetamine, hydrocodone, fentanyl, and hydromorphone. When the groups were separated, among patients who had CAD, the first three prescription opioid medication groups: CONTSUB 1, 2 and 3 were found to be statistically significant (χ^2^ = 25.016, *p* < 0.001, χ^2^ = 15.193, P < 0.001, and χ^2^ = 6.553, *p* = 0.010 respectively).

### 3.4. Co-Morbidities: Hypertension, Hyperlipidemia, Diabetes Mellitus, and Obesity

Among patients who have CAD, 1268 (69.3%) of them reported that they have been diagnosed with high blood pressure. There was a strong association (*p* < 0.001) between hypertension and coronary artery diseases and myocardial infarction. More than half of the patients 963 (52.7%) who had the coronary artery disease have hyperlipidemia, and hyperlipidemia was also significantly associated with CAD (*p* < 0.001). Similarly, among these patients, 352 (19.2%) had diabetes and 232 (12.7%) of them were obese.

### 3.5. Alcohol Misuse, Abuse or Dependence, and Substance Abuse

Although only 1.1% (21) of patients included in the study were heavily dependent on alcohol, it was still strongly associated (0.015) with coronary artery disease. This is similar to the results among patients who abused substances (3.0% (55), *p* = 0.010).

### 3.6. Logistic Regression

In Table 3, the results of the binary logistic regression analysis are presented. A forward Wald stepwise method was used to build the model. The dependent variable was CAD (coronary artery disease), the predictors of interest was the prescription opioid variables whose p-values were less than 0.05 from the initial chi-square performed, this was CONTSUB 1-3. Covariates included were age, race/ethnicity, sex, and comorbidities of diabetes mellitus, hyperlipidemia, and hypertension.

Age categories 25–44, 45–64, 65–74, and ≥75 were all significantly associated with the dependent variable; coronary artery disease [(*p* < 0.001), 95% CI: 0.00–0.12, 0.04–0.11, 0.25–0.34, and 0.55–0.74 respectively). Cardiovascular disease was significantly associated with the female sex [(*p* < 0.001), 95% CI: 0.40–0.51]. For race/ethnicity, the Prevalence Odds Ratio (POR) for Non-Hispanic Whites was 1.14 (95% CI: 0.79–1.65); and for Hispanics, POR 1.06 (95% CI: 0.68-1.64). For Non-Hispanic Black, POR 0.978 (95% CI: 0.65–1.48). These prevalence odds ratios suggest that no association exists between these variables.

For comorbidities, type 2 diabetes mellitus was a significant predictor of the dependent variable coronary artery disease [(*p* < 0.001), POR: 0.66, 95% CI: 0.57–0.78]. This is similar to the results obtained for hyperlipidemia [(*p* < 0.001), POR: 0.38, 95% CI: 0.34–0.44], and hypertension [(*p* < 0.001), POR: 0.51, 95% CI: 0.45–0.59]. For the predictors of interest, compared to medications in “other schedule”, prescription opioids codes 1–3 did not show significant association; controlled substance code 1: *p* = 0.34, POR: 1.39, 95%CI: 0.71–2.75; substance code 2: *p* = 0.59, POR: 1.20, 95% CI: 0.61–2.37, and controlled substance code 3: *p* = 0.62, POR: 0.85, 95%CI: 0.45–1.6.

## 4. Discussion

Cardiovascular diseases are the top causes of deaths in the United States. In the 2015 NAMCS data, 6.5% of the patients had coronary artery disease or MI. Some studies have predicted that amongst other already known risk factors for cardiovascular diseases, use of opioid may be a novel risk factor. We further explored this to understand whether use of prescription opioid medication was associated with coronary artery diseases among patients in the 2015 NAMCS.

In the present study, there was no statistically significant association found between prescription opioid medication use and coronary disease. However, some studies have found contrasting results, with evidence of acute cardiac arrest with opioid use, especially in cases of overdose [16,17]. A more in-depth study that looked at the possible association between extended release oxycodone-naloxone therapy and ischemic cerebrovascular events also found no direct association, although the study prescribed the need for more research in this area [18].

Other studies have explored specific opioid medication, such as methadone. Studies have looked at the cardiotoxicity of methadone, and after finding some significant results, they recommended a review of the prescription guideline of methadone among patients with cardiovascular compromise [17,19]. Other studies similar to the current study have also submitted that both short-term and long-term opioid use did not significantly increase the risk of heart disease [5,20]. When reviewing the current literature, some studies suggested that long-term opioid medication may have very little direct effects on the functions of the heart. These results were considered rather inconclusive by these authors [6].

For cardio-protectiveness, analyses from the current study does not suggest that opioid pain medications conferred protectiveness, although results from some studies have found evidence that opioid medications may have some measure of cardio-protective properties [21]. For morphine specifically, a study found that this medication was very useful in the opioidergic conditioning of the heart [22]. This is especially so since morphine is one of the important emergency medications for acute myocardial infarction.

Due to their many potential adverse effects, opioid medications are used with care among high-risk patients, such as persons with cardiovascular diseases, liver and renal compromise, elderly and younger patients, pregnant women etc. For pregnant women, it is important to note that in the United States, cardiovascular diseases are the leading cause of maternal deaths in this population [23]. A study that analyzed the trends of opioid use and cardiovascular-related deaths among pregnant women found a positive association between the two. However, analysis from the current study does not include data on pregnant women; hence this study did not explore separately whether an association exists between opioid use and cardiovascular disease in this group.

Several other classes of medications have been implicated in the development of cardiovascular diseases. One of such groups is Non-Steroidal Anti-inflammatory Drugs (NSAIDS). In fact, Non-Steroidal Anti-inflammatory Drugs, when consistently used in high doses, have been implicated in increases in high blood pressure. The rationale for these adverse effects lies in the effects of these mediation class on renal function, development of edema. NSAIDs have been shown to have an untoward effect on the cardiovascular system, with an increased risk of stroke and myocardial infarction, including gastrointestinal bleeding [24,25].

The results from this study further show the need for more research in this area. Although, a direct association was not elicited, however, some categories of the opioid groups analyzed in the study showed results that may suggest cardio-protectiveness. Subsequent subgroup analysis will explore the cardio-protectiveness of morphine as observed in a previous study [22]. Other specific opioid medicines (acetaminophen-oxycodone, oxycodone, amphetamine-dextroamphetamine, hydrocodone, fentanyl, and hydromorphone) reported by participants will also be analyzed in subsequent studies. The results remain conflicting as to the effects that opioid therapy have on the cardiovascular system of adults.

## 5. Limitations of the Study

In the current study, data from only one survey year (2015) was included. Subsequently, for reliability and further insights, future studies need weighted data from more than one year or survey period. The NAMCS survey is considered nationally representative, and the sampling method was redesigned in 2012 to include state estimates, for the 34 most populous state in the United States, and the nine census divisions [25]. However, non-response bias in NAMCS has been identified and is usually taken into consideration during analysis.

The study design also presents several limitations. As a cross-sectional study, the temporal order of the exposure and the outcome cannot be sufficiently determined, hence we interpreted the results as whether an association existed rather than an attempt to prove causality. This reason also limits the interpretation of the prevalence odds ratio in this study. We recognize existing information bias; the limitations of self-report of the presence of the diseases, comorbidities, and we did not have information on the doses, compliance, misuse and duration of use of prescription opioid medicines. The observational design also raises the question of reverse causality since the temporal order of exposure to prescription opioids and presence of CVD cannot be determined. We could argue that except in cases of cardiovascular events such as an acute MI, opioids medications are not typically used to manage CVD, however, lack of information about the duration of opioids use and the reasons for use obscures this argument.

## 6. Conclusions

Cardiovascular diseases and their interaction with opioid use remains an intriguing research area; we could not convincingly define this nexus. Known risk factors of hypertension, hyperlipidemia, diabetes mellitus, obesity, and lifestyle risks of alcohol use, smoking and substance abuse were strongly associated with coronary artery disease. Our findings also suggested that some association may exist between cardiovascular diseases and some specified opioid medications, rather than grouped class of medicines. We intend to explore this in subsequent studies.

## Figures and Tables

**Table 1:** Characteristics of the sample population.

Count, *n*, (%)	Coronary Artery Disease (CAD) (*N* = 1829)	Chi Square Value	*p*-Value
Age, years		χ^2^ = 1899.874	*p* < 0.001 ^a^
Under 15	4 (0.2%)		
15–24	1 (0.1%)		
25–44	32 (1.7%)		
45–64	392 (21.4%)		
65–74	604 (33%)		
≥75	796 (43.5%)		
Sex		χ^2^ = 272.615	*p* < 0.001 ^a^
Male	1147 (63%)		
Female	682 (37%)		
Race/ethnicity		χ^2^ = 73.012	*p* < 0.001 ^a^
Non-Hispanic White	1536 (84%)		
Non-Hispanic Black	134 (7.3%)		
Hispanic	107 (5.9%)		
Non-Hispanic Other	52 (2.8%)		
Tobacco use		χ^2^ = 54.216	*p* < 0.001 ^a^
Not Current	1431 (78.2%)		
Current	189 (10.3%)		
Never Use	669 (35.3%)		
Former	645 (35.3%)		
Body Mass Index		χ^2^ = 392.570	*p* < 0.001 ^a^
Underweight <18.5	554 (30.7%)		
Normal 18.5–24.9	280 (15.5%)		
Overweight 25.0–29.9	425 (23.5%)		
Class I Obesity 30.5–34.9	315 (17.5%)		
Class II Obesity 35.0–39.9	134 (7.4%)		
Class III Obesity ≥40.0	97 (5.4%)		

^a^*p* = 0.000, ^b^ *p* < 0.05. Date source Center for Diseases Control and Prevention. National Ambulatory Health Care Data: Data Sets and Documentation. (November 30, 2017). Accessed September 27, 2018. https://www.cdc.gov/nchs/ahcd/datasets_documentation_related.htm [13].

**Table 2:** Chi-square test of independence for certain risk factors of Coronary Artery Disease (CAD).

Variables, *n*, (%)	Coronary Artery Disease (CAD) (*N* = 1829)	Chi-Square Value	*p*-Value
CONTSUB 1		χ^2^ = 25.016	*p* < 0.001 ^a^
Schedule II	14 (0.9%)		
Other Schedules ^c^	1594 (99.1%)		
CONTSUB 2		χ^2^ = 15.193	*p* < 0.001 ^a^
Schedule II	10 (0.7%)		
Other Schedules	1442 (99.3%)		
CONTSUB 3		χ^2^ = 6.553	*p* = 0.010 ^b^
Schedule II	12 (0.9%)		
Other Schedules	1358 (99.1%)		
Alcohol misuse, abuse or dependence	21 (1.1%)	χ^2^ = 5.927	*p* = 0.015 ^b^
Type 2 Diabetes	352 (19.2%)	χ^2^ =539.858	*p* < 0.001 ^a^
Hyperlipidemia	963 (52.7%)	χ^2^ = 1943.517	*p* < 0.001 ^a^
Hypertension	1268 (69.3%)	χ^2^ = 1777.115	*p* < 0.001 ^a^
Obesity	232 (12.7%)	χ^2^ = 123.778	*p* < 0.001 ^a^
Substance Abuse	55 (3.0%)	χ^2^ = 6.637	*p* = 0.010 ^b^

^a^*p* = 0.000, ^b^ *p* < 0.05, ^c^ Other Schedules contained controlled substances schedules III, IV, and V as classified by the Controlled Substances Act (CSA). Date source: Center for Diseases Control and Prevention. National Ambulatory Health Care Data: Data Sets and Documentation. (November 30, 2017). Accessed September 27, 2018. https://www.cdc.gov/nchs/ahcd/datasets_documentation_related.htm [13].

**Table 3:** Logistic regression analysis results.

Variables	B	S.E. B	Wald	*p* Value	POR	95% CI POR	
Patient age							
25–44	−4.080	1.004	16.514	0.000	0.017	0.002	0.121
45–64	−2.738	0.252	118.37	0.000	0.065	0.040	0.106
65–74	−1.249	0.081	237.58	0.000	0.287	0.245	0.336
≥75	−0.449	0.073	38.321	0.000	0.638	0.553	0.736
Race/ethnicity							
Non-Hispanic White	0.135	0.187	0.520	0.471	1.144	0.794	1.649
Non-Hispanic Black	−0.022	0.213	0.011	0.918	0.978	0.645	1.484
Hispanic	0.058	0.223	0.067	0.795	1.060	0.684	1.641
Patient sex (reference group - female)	−0.797	0.064	156.93	0.000	.451	0.398	0.510
Diabetes mellitus Type 2	−0.413	0.080	26.363	0.000	.662	0.565	0.775
Hyperlipidemia	−0.958	0.065	218.50	0.000	0.384	0.338	0.436
Hypertension	−0.666	0.070	90.793	0.000	0.514	0.448	0.589
Controlled substance code for medication #1	0.332	0.347	0.915	0.339	1.394	0.706	2.751
Controlled substance code for medication #2	0.186	0.345	0.291	0.590	1.205	0.612	2.371
Controlled substance code for medication #3	−0.159	0.324	0.240	0.624	0.853	0.453	1.609
Constant	−0.177	0.599	0.087	0.768	0.838		

Date source: Center for Diseases Control and Prevention. National Ambulatory Health Care Data: Data Sets and Documentation. (November 30, 2017). Accessed September 27, 2018. https://www.cdc.gov/nchs/ahcd/datasets_documentation_related.htm [13].
